# Special Issue of Environment-Friendly Construction Materials

**DOI:** 10.3390/ma12071101

**Published:** 2019-04-03

**Authors:** Shaopeng Wu, Inge Hoff, Serji Amirkhanian, Yue Xiao

**Affiliations:** 1 State Key Laboratory of Silicate Materials for Architectures, Wuhan University of Technology, Wuhan 430070, China; wusp@whut.edu.cn; 2 Department of Civil and Environmental Engineering, Norwegian University of Science and Technology, Hogskoleringen 7A, NO-7491 Trondheim, Norway; Inge.Hoff@ntnu.no; 3 Department of Civil Construction and Environmental Engineering, University of Alabama, Tuscaloosa, AL 35487, USA; samirkhanian@eng.ua.edu

**Keywords:** construction materials, fatigue life, ageing resistance, modified asphalt materials, rejuvenator, self-healing asphalt, recycling, cold recycled asphalt mixture, ultra-high performance concrete

## Abstract

This special issue, “Environment-Friendly Construction Materials”, has been proposed and organized as a means to present recent developments in the field of construction materials. For this reason, the articles highlighted in this editorial relate to different aspects of construction materials, from pavement materials to building materials, from material design to structural design, from self-healing to cold recycling, from asphalt mixture to cement concrete.

Construction materials are the most widely used materials for civil infrastructures in our daily life. However, from an environmental point of view, they consume a huge amount of natural resources and generate the majority of greenhouse gasses. Therefore, many new and novel technologies for designing environment-friendly construction materials have been developed recently. This special issue, “Environment-Friendly Construction Materials”, has been proposed and organized as a means to present recent developments in the field of construction materials. It covers a wide range of selected topics on construction materials. A brief summary of the articles is given in this editorial.

Service life prediction is essentially important in designing construction materials. Researchers all over the world are devoting themselves to life prediction analysis. Sun et al. [1,2] used a plateau value and permanent deformation ratio from three-point bending fatigue tests with cyclic loading to predict the fatigue life of as asphalt mixture. The fatigue equation based on a plateau value can well predict the fatigue life. Wang et al. [3] studied the fatigue performance of combined structures with hot mix asphalt and cement emulsified asphalt mixtures. An artificial neural network was used and fatigue equations were established for fatigue life prediction. Residual fatigue properties of asphalt pavement after long-term field service [4], low-temperature performance [5] and damage characteristics [6] were reported. Eco-friendly fiber was used to improve the performance of mixtures. Sun et al. [7] studied the viscoelastic mechanical responses of high-modulus asphalt pavement by numerical simulation with a moving load. In three articles, ageing resistances of asphalt were reported, including ageing depth resulting from ultraviolet radiation [8], fluorescence spectrum ageing analysis [9], and ageing improvement by SBS/CRP (Styrene–butadiene–styrene polymer/crumb rubber powder) modification [10]. One research article focused on the chemical evolution and rheological properties of asphalt under water solute exposure [11]. Saturates and aromatics were partly dissolved in water and then moved out.

Modification on construction materials are being conducted in many research institutes to design durable civil infrastructures. Fiber is a widely used strengthening additive in asphalt mixtures. Eco–friendly basalt fiber was incorporated with SBS and diatomite by Wang et al. [12] and Cheng et al. [13]. Another article by Yang et al. [14] presented improving mechanisms of diatomite modified asphalt mixtures, by means of permanent deformation resistance and moisture resistance. Aluminum hydroxide and layered double hydroxide were proposed by Li et al. [15] to improve the fire resistance of asphalt. Another nanomaterial, named nanosilica, was evaluated by Guo et al. [16]. 

Improving the aggregate morphology characteristics is another effective way to get durable asphalt mixtures. Xiao et al. [17] established the relationship between fine aggregate morphology and skid-resistance of micro-surfacing, while Cheng et al. [18] and Wang et al. [19] reported the influence of aggregate morphological characteristics on asphalt mixtures. The studied aggregate morphological characteristics include roundness, perimeter index, erosion-dilation area ratio, angularity, and surface texture. Influence of aggregate characteristics on the demulsification speed of asphalt emulsion was presented by Tang et al. [20]. Furthermore, Liu et al. [21] proposed to use ash byproduct to improve the asphalt-aggregate adhesion properties.

Rejuvenator, a healing agent to recover aged asphalt binder, is a widely used material in pavement preventive maintenance. There are many different rejuvenators. For instance, soybean oil based [22], dodecyl benzene sulfonic acid based [23], bio-oil based [24], petroleum based [25], isocyanate and epoxy substances based [26] were detailed in this special issue. The interesting rejuvenation enhancement was investigated and reported by these articles. Healing behavior of asphalt materials is another key issue in the pavement preventive maintenance. Calcium alginate capsules were designed by both Xu et al. [27] and Shu et al. [28]. The former article investigated the healing capacity of asphalt mixture when calcium alginate capsules were used, while the second article presented a preparation process for calcium alginate capsules with a multinuclear structure. In the study by Wan et al. [29], self-healing properties of steel fiber and steel slag based ultra-thin wearing course were studied by a semi-circular bending test under induction heating. Other researches focused on the healing agent effect [30], induced healing efficiency of induction heating and microwave heating [31], and initial self-healing temperature [32].

Andrzejuk et al. [33] and Ogrodnik et al. [34] reported their research on reusing the wastes of sanitary ceramics as aggregates for asphalt mixture and cement concrete, respectively. Waste concrete powder [35], low-grade aggregate [36], crumb rubber waste [37], and recycled concrete aggregate [38] were also successfully reused as construction materials. In the study by Li et al. [39], the reclaimed asphalt pavement was reused 100% in cold recycled asphalt mixtures. Asphalt emulsion and cement were used to improve the interfacial bonding between binders and fillers, aiming to enhance the moisture resistance and high temperature stability.

Several other studies involved the evaluation of eco-friendly railway concrete sleepers [40] and engineered cementitious composites [41]. In the research of the former article, waste rubber was reused for high-strength rubberized concrete. It was found that a decrease of compressive strength can be expected when rubber content increased, and 10% was recommended as the optimal reuse content. In the latter article, modified polyvinyl alcohol fiber was added into engineered cementitious composites to enhance the mechanical performance. Research on self-compacting concrete [42], ultra-high performance concrete [43], cement paste plasticized by polycarboxylate superplasticizer [44], and pozzolanic additive in cement [45] were also discussed in this special issue.

Last, but not least, there are two articles focusing on functional construction materials, like phase change materials for building energy conservation [46], and graphene-modulated removal performance of nitrogen and phosphorus pollutants [47].
